# Liquidity constraints, cash transfers and the demand for health care in the Covid‐19 pandemic

**DOI:** 10.1002/hec.4585

**Published:** 2022-08-22

**Authors:** Carlos Alberto Belchior, Yara Gomes

**Affiliations:** ^1^ University of Brasília Brasilia Distrito Federal Brazil; ^2^ University of Zurich Zurich Switzerland; ^3^ Federal University of Pernambuco Recife Pernambuco Brazil

**Keywords:** auxílio emergencial, Covid‐19, demand for medical services, liquidity constraint

## Abstract

This paper proposes the hypothesis that liquidity constraints may delay or even prevent sick individuals from seeking medical help. If this is the case, a cash transfer can directly increase the demand for medical care. We evaluated this hypothesis empirically in the context of the implementation of Emergency Aid (EA), a large‐scale cash transfer program in Brazil, during the Covid‐19 pandemic. We used the program's implementation calendar along with a Regression Discontinuity in Time to assess the causal effects of EA on the search for the health system. Consistent with our hypothesis, we estimate that the transfer immediately decreased the time to search for the health system by 14% and increased COVID‐19 hospitalizations by 0.015%.

## 1 INTRODUCTION

In the last decade, we observed four pandemics emerge throughout the world, which posed considerable risk to individuals and substantial challenges to governments. In this paper, we examine the existence of “liquidity sensitivity” behavior in the most recent pandemic (COVID‐19), that is, whether sick individuals delay healthcare consumption due to lack of income and access to credit markets.

Consider, for instance, an informal worker who depends on their daily earnings to provide for their family. Even if they are infected, the lack of income might prevent them from searching the healthcare system. This behavior might not only aggravate the individuals' disease but also increase the probability of them infecting others.

We evaluate this hypothesis in the context of the Emergency Aid (EA) program in Brazil, a large cash transfer program undertaken during the COVID‐19 pandemic. We evaluate if the program, that increased beneficiaries' liquidity, affected their healthcare consumption either by increasing COVID‐19‐related hospitalizations or decreasing the lag between becoming ill and accessing the healthcare system.

To test this hypothesis, we take advantage of the fact that different birth cohorts received the transfer on different days and that the transfer should increase beneficiaries' healthcare consumption *immediately* if they are liquidity constrained. We exploit these factors by implementing a regression discontinuity in time (RDiT) design.

We find that the treatment reduced the lag in the search for the health system by 14%. Back‐of‐the‐envelope calculations suggest that the reduction in the delay to search for the healthcare system decreased the number of new daily infections by 2450 (i.e., almost 20% of the total number of infections at the time). We also find that the *EA* increased the number of hospitalizations by 0.015%, which amounts to 400 new hospitalizations in the 5 days immediately following the transfer. This increase in hospitalizations suggests that even individuals who had severe symptoms (such that immediate hospitalization was necessary) were liquidity‐ and income‐constrained, which prevented them from reaching the healthcare system.

We show that these results are robust to the use of different regression discontinuity approaches, such as a sharp regression discontinuity in time and a local randomization approach. We also take advantage that different cohorts received the payment at different points in time to test potential spillovers and anticipation effects. We find that the demand for healthcare for cohorts that receive the money later does not change once other cohorts are paid, but only when these cohorts actually receive the money.

We contribute to three strands of literature. First, we contribute to the very recent literature that documents similar “liquidity sensitivity” in health care consumption (Gross et al., 2022; Lyngse, 2020). We add to it by documenting changes in another measure of healthcare consumption, other than prescription drugs. We also provide additional evidence that corroborates the fact that liquidity constraint is the relevant mechanism for the observed change in behavior. We show that the *EA* not only increased healthcare consumption but also *immediately* decreased sick individuals' delay in accessing necessary care. This is not consistent with alternative explanations such as the indirect health effects deriving from other changes in consumption (Gross & Tabacman, 2014).

The context of our paper also differs from previous ones in some other interesting ways. We analyze a developing country with free healthcare, and therefore, our results suggest that individuals are not able to give up their daily earnings to reach the health system. Also, in our context, delaying medical care might generate negative externalities.

Second, we contribute to the literature that examines the direct effect of cash transfers or exogenous income increases on health (Apouey & Clark, 2015; Cawley et al., 2010; Dobkin & Puller, 2007; Lindahl, 2005; Van Kippersluis & Galama, 2013). These papers emphasize only the effects on health, generated by changes in consumption patterns and usually estimate negative effects of positive income shocks on health. We focus on a different mechanism, that is, the demand for healthcare and show that these transfers might not only have positive effects on the beneficiaries' but also generate positive externalities on the health of others.

Third, we contribute to the literature that analyzes the impacts of cash transfers during the pandemic. So far, the studies on this topic have focused on the impacts of the transfers on consumption (Casado et al., 2020; Coibion et al., 2020), poverty (Ree‐Jones et al., 2020), and preferences for the size of the state safety net (Han et al., 2020). We contribute to this literature by documenting one potential benefit of cash transfer that has not yet been emphasized, that is, its direct impact on the demand for healthcare.

This paper is divided into five sections other than this introduction. Section 2 describes the Brazilian healthcare system, the context of the COVID‐19 pandemic in Brazil, and the Emergency Aid program. Section 3 describes data sources and the sample of the study and provides some descriptive statistics. Section 4 presents our econometric approach, discusses identification and inference, shows our main results, and discusses potential mechanisms. Section 5 details the robustness checks we carried out. Finally, Section 6 concludes the paper.

## 2 INSTITUTIONAL BACKGROUND

### 2.1 The healthcare system and the COVID‐19 pandemic in Brazil

Brazil has a large unified public healthcare system (SUS) that is committed to providing integral health coverage, including preventive care, medical appointments, medicine, and hospitalizations. During the COVID‐19 pandemic, all individuals could be tested and receive needed treatment for free in SUS. Alternatively, individuals could pay and receive medical treatment in the private facilities. It is estimated that SUS caters to three‐quarters of the Brazilian population (Bahia & Scheffer, 2018). The Brazilian health system is regarded as advanced, compared to its neighbors and other middle‐income countries (Castro et al., 2019). However, SUS was overwhelmed during the pandemic.

The COVID‐19 pandemic hit Brazil particularly hard. Despite the first registered case being recorded relatively late, on February 26, 2020, the country could not effectivy enforce containment measures and the contagion spread with dramatic speed. Until April 2022, Brazil reported approximately 660 thousand deaths by COVID‐19, which amounts to more than 3000 deaths per million population. This is the 2nd largest COVID‐19 death toll in the world (behind only the United States) in terms of absolute numbers and 15th largest after taking into account the total population.

To fight the pandemic, Brazilian states implemented a lockdown in the second week of March 2020. A package of different economic measures was rolled out to enable adherence to non‐pharmacological measures and countervail the macroeconomic and welfare‐related effects of the pandemic. These measures include extended unemployment insurance, expansion of the credit offer and income transfers. The most important of these economic measures was the Emergency Aid (EA), which we describe below in detail.

### 2.2 The emergency aid (EA) program

The *EA* program was a direct and unconditional cash transfer to unemployed individuals or informal market employees to ensure income for these vulnerable individuals while pandemic containment measures were in force. Initially, the government planned the *EA* as a 3‐month program, but it was extended twice in 2020 because of the escalation of the pandemic. In 2021, it was extended again. In the end, the program was implemented from April 2020 to November 2021.

To be eligible for, an unemployed person or informal market worker needed to be age 18 years or more, not a recipient of any other assistance from the government, such as unemployment insurance. An exception was made for the Bolsa Família (BF) program, in that case, the recipient would receive the eligible benefit, with the higher payment. Also, the recipient's monthly per capita family income could neither be higher than half of the minimum wage, R$522,50 (US$107,55), nor could the total family income exceed R$3.135,00 (US$645,3) (IFI, 2020).

To apply for being a beneficiary under the program, individuals had to fill a program‐specific application with their personal information. The government then verified whether the information provided in the application matched with public records or not and decided if the applicant was eligible for the *EA* payout.

The *EA* provided a monthly transfer between R$600,00 (US$123.50) and R$1.200,00 (US$ 265.00), depending on the applicant's family composition. Single‐parent women were eligible for the R$1.200,00 (US$265.00) benefit (IFI, 2020). The value of the transfer was relatively generous, as the standard benefit represented approximately 40% of the per capita household income, 60% of the minimum wage, and about twice the maximum benefit granted by other income transfer programs.^1^

The program had significant penetration, with 66 million individuals and half of the Brazilian households benefiting from the transfer (Duque, 2020). The three initial payments of *EA* were estimated to cost between R$134,6 (US$16,62) and R$154,4 (US$31,8) billion. The transfers started being handed out in April 2020. To minimize agglomerations on bank branches, installments were not transferred to all individuals simultaneously. Table 1 shows the schedule for the first payment.

**TABLE 1:** Aid availability dates

Cohort	Month of birth	Availability dates
1	Jan/Feb	04/27
2	Mar/Apr	04/28
3	May/Jun	04/29
4	Jul/Aug	04/30
5	Sep/Oct	05/04
6	Nov/Dec	05/05

*Note*: Availability dates of the first emergency aid installment for different birth cohorts.

This calendar will be used in our empirical strategy. As discussed above, the program was announced before its launch and individuals had to register and be declared eligible. Thus, individuals received income shocks in the availability dates, but no additional information. We focused on the first emergency aid payment because after the first one, several parallel schedules were generated with overlapping payment dates, which prevents the expansion of our analysis.

It is important to note that increasing the health care demand wasn't one of the *EA* program's laid down objectives. The easing of the liquidity constraint and the consequent increase in health care demand were the program's unintended positive consequences.

## 3 DATA AND SAMPLE

### 3.1 Data

We used two main data sources, covering the period between April 4 and July 25, 2020.

#### 3.1.1 Hospitalizations data

Microdata of all kinds of hospitalizations for respiratory syndromes in the public health system. It also included detailed information about patients and medical procedures available for the universe of hospitalizations. We are interested in the number of daily COVID‐19‐related hospitalizations in each municipality. In Appendix B we provide descriptive statistics for the COVID‐19 related hospitalizations in the sample.

#### 3.1.2 Tests data

National microdata for COVID‐19 tests, generated by local health units. Both tests and individuals' characteristics are available. We are interested in the average time that elapsed between the first symptoms, reported by the individual and the test date for each municipality. In Brazil, the COVID‐19 self‐tests were regularized by the Brazilian Health Regulatory Agency (Anvisa) at the beginning of 2022. Until then, self‐tests were not directly available to the population. Instead, individuals who wanted to get tested were required to go to hospitals, drugstores, or other health facilities. These facilities were required to report the test results and individuals' personal characteristics to the local health authority. Thus, our sample incorporates the almost all of the tests conducted in Brazil during the said the period.

The data on both hospitalizations and tests were provided by the health facilities and gathered by the Health Ministry. We also use complementary data on access to the banking system. The Brazilian central bank has the location of all of the bank branches in the country. We scraped this information from the central bank's website and used the municipalities' number of bank branches as a proxy for individuals' access to the bank system. We use this data to provide additional ancillary evidence in favor of the liquidity constraint hypothesis, detailed in Appendix C.

### 3.2 Sample and descriptive statistics

For our empirical analysis, we are interested in two variables. The first one is the average time elapsed between the first symptoms and the search for the health system. The date on which individuals were tested were used as a proxy for the time they reached the health system, as this is usually the first procedure to be adopted. We consider this to be a very broad measure of the demand for healthcare during the pandemic.

The second variable of interest is the number of COVID‐19 related hospitalizations. We consider these to be a more stringent measure of the demand for healthcare. The likelihood of an infected individual being hospitalized is likely to be positively correlated with the severity of the disease. This second variable of interest may capture the intensity of the problem of liquidity constraints. Even if individuals who needed immediate hospitalizations were constrained not to search for the healthcare system, this only points to the seriousness of the problem and its potential impact on poorer individuals. Table S2 in the Appendix B of the Supplementary Materials shows descriptive statistics, for individual‐level hospitalizations.

We include in the sample all of the registered positive COVID‐19 tests and related hospitalizations for working‐age adults and aggregated these variables in three dimensions: municipalities, cohorts, and days. The lag in the demand for healthcare is available when at least one COVID‐19 case was registered. We also add one and apply the log transformation to the total number of hospitalizations. This procedure allows us to interpret the treatment effects as approximate semi‐elasticities and does not require us to drop observations with zero hospitalizations. In Appendix C, we show that our results are robust to other similar transformations in the dependent variable. Table 2 presents the descriptive statistics for our aggregate sample.

**TABLE 2:** Descriptive statistics

	Average	Standard‐deviation	Minimum	Maximum
Municipalities/day				
Hospitalizations	0.07	1.11	0.00	111
Deaths	0.02	0.39	0.00	48
Days to test	9.48	9.13	0.00	146
Population (thousands)	39.45	227.64	0.84	12,325.232
Observations				
Municipalities (A)	5334			
Cohorts (B)	6			
Days (C)	120			
Observations (A*B*C)	3,872,484			

*Note*: Descriptive statistics for aggregate variables of interest at the municipality, cohort, and day level. Total number of observations is given by multiplying municipalities, cohorts and days.

We have information for the universe of hospitalizations in the public system, but not from the private system. This restriction decreases the generality of the sample, but we do not consider this to be a significant constraint, for as mentioned above, the public health system caters to the vast majority of the Brazilian population (Bahia & Scheffer, 2018). Also, given that we are interested in individuals in situations of economic vulnerability, the potential variation in hospitalizations should be almost universally concentrated in the public health system.

## 4 EMPIRICAL ANALYSIS

### 4.1 Regression discontinuity in time (RDiT)

To evaluate the impact of the *EA* on healthcare consumption, we rely on a Regression Discontinuity in time (RDiT) specification. Our favorite specification is:

(1) $$y_{\mathrm{mct}}=\alpha +\beta *1\left[d_{t}-EA_{c}>0\right]+\lambda f\left(d_{t}\right)+\delta X_{\mathrm{mct}}+\psi_{m}+\epsilon_{\mathrm{mct}}$$

where *y*_*mct*_ is the outcome for municipality *m*, cohort *c* and day *t*, **1**[.] is an indicator function, *d*_*t*_ is the calendar day; *EA*_*c*_ is the day cohort *c* receives the transfer, *f*(.) is a flexible function, ***X***_*mct*_ are weekday dummies and *ψ*_*m*_ are municipalities fixed‐effects. We follow Hausman and Rapson (2018) in our favorite specification, using a linear function on each side of the cutoff, uniform kernel, and a 5‐day bandwidth, which is close to the one estimated using Calonico, Cattaneo, and Farrell's (2020) method.

The fundamental identification hypothesis in the equation above is that the error term distribution is continuous around the cutoff. In this case, *β*, recovers the causal effect of the *EA* on the variable of interest. Intuitively, we need to assume that non‐observable confounders do not change discontinuously at the same time the *EA* is given.

When analyzing hospitalizations, we use a log specification combined with municipality fixed‐effects as shown in Equation (1). The intuition for this regression is that we are estimating the discontinuity in hospitalizations in percentage points within each municipality and then aggregating the estimated change in hospitalizations for the whole sample. Therefore, we are controlling for any fixed municipality characteristic, such as population.^2^

As a robustness check, we can also take advantage of the discontinuities generated by the program to estimate treatment effects in another way. Instead of relying on the sharp RDiT design described above, we can implement a local randomization regression discontinuity. In this approach, we assume that the treatment assignment is effectively random at a *very* narrow window around the cutoff. If this is the case, we can estimate the effect of the *EA* on the demand for healthcare by estimating:

(2) $$y_{\mathrm{mct}}=\alpha +\beta *1\left[d_{t}-EA_{c}>0\right]+\epsilon_{\mathrm{mct}}$$

that is, by simply comparing the averages of the demand for healthcare immediately before and after the transfer is made by individuals. To make this approach more plausible, we focus on the smallest possible window around the cutoff, just 1 day before and 1 day after the transfer was made.

There are two potential advantages in using the local randomization approach relative to the sharp RDiT (Cattaneo et al., 2019). First, it is more direct and transparent and we do not have to rely on any covariates (such as weekdays fixed‐effects) for identification. By focusing on a 2‐day window, we restrict the potential effects of seasonality. Second, in applications in which the running variable is discrete and there are few mass points (as in our case), it might be difficult to control for *f*(*d*_*t*_).

The regression discontinuities approaches mentioned above are not the only feasible empirical strategy in our context. Alternatively, we could estimate a difference‐in‐difference (DiD) model. However, there are two reasons that makes us opt for the RDiT as our main strategy. The first one is that the RDiT approximates the effect of the treatment on the outcome of interest just around the cutoff, while the DiD model recovers a weighted average of the cash transfer in the post‐treatment period. As discussed above, if individuals are liquidity constrained, we expect to observe an instantaneous effect on the demand for healthcare. Thus, the RDiT provides a better approximation for the theoretical effect we have in mind.

The second reason is that in the DiD model we would need to assume parallel trends in the demand for healthcare across birth cohorts. Recent investigation has shown that the month of birth tends to be highly correlated with family characteristics (Buckles & Hungerman, 2013). The baseline differences across groups tend to decrease the plausibility of the identifying assumption, as the same mechanism that generates these differences may generate different trends across groups (Khan‐Lang & Lang, 2020). In the RDiT design we do not need to impose restrictions on the joint distribution of the dependent variables *across* cohorts, but only on the continuity of the error‐term *for each* cohort. Therefore, we think that, in our context, the identifying assumption for the RDiT is weaker than for the DiD. Nonetheless, in Appendix C, we show results for the DiD model and find that our estimates remain essentially unchanged even if we estimate a difference‐in‐difference model.

### 4.2 Identification and inference

The RDiT framework does generate specific difficulties for identification and inference that usually do not arise in the cross‐sectional RDD (Hausman & David, 2018). These include the following: (1) impossibility of sorting tests (Bugni & Canay, 2019; McCrary, 2008); (2) the frequent need to use a large window of observations, (3) time dependence of the error‐term, (4) the need to model time‐heterogeneous treatment effects, and (5) the need to include covariates in the estimation.

The first concern is not particularly important for our application. The aid implementation was strict in terms of preventing the money being available to beneficiaries before the official release day, seeking to avoid agglomerations. We also test the presence of sorting around the cutoff using the different timing cohorts that received treatment as detailed in Section 5.

Second, since we do not depend exclusively on time‐series variation, we can use a very restrictive bandwidth of only 5 days in our favorite specification, which decreases the likelihood of the time‐variant confounders affect our estimates and allows us to focus on the immediate period after the cash transfer is made, where we expect liquidity constraint's effects to be concentrated. Also, in the local randomization approach, we use the most narrow possible window, only 1 day on each side of the cutoff, restricting even further the possibility of time‐varying confounders playing any role. Third, to deal with the time‐dependent error terms, we present standard errors clustered in two dimensions,^3^ day and municipality level.

Fourth, we can make strict predictions about time heterogeneity. We expect the treatment to have a positive immediate effect on hospitalizations. However, besides alleviating liquidity constraints, the transfer allows individuals to protect themselves against the disease, which decreases the total number of contagions. We expect this “protection” effect to generate a downward trend in hospitalizations' treatment effects after 5 days of the transfer,that is, the Sars‐CoV‐2's incubation time (Backer et al., 2020). As for the lag in search for the healthcare system, we expect it to be negative, independent of the time horizon. We discuss this time‐heterogeneity patterns in Appendix A of the supplementary materials.

Finally, unlike the standard cross‐sectional regression discontinuity design, in the RDiT, the identification hypothesis of the error‐term continuous distribution frequently is only plausible after conditioning on covariates. To see why, let us focus on the first cohort, that received the payment on April 27, 2020, a Monday. Our empirical strategy compares the demand for health in the days just after the transfer with the demand on the days immediately before. We claim that it is highly unlikely that other structural determinants of demand change discontinuously in such a narrow time frame. However, the demand for healthcare variables is seasonal (hospitalizations, for instance, are usually higher on the weekend) and this pattern risks confounding the effects of the treatment. The fact that cohorts started receiving treatment on different days helps attenuate concerns with seasonality, but we also include weekday fixed‐effects to provide additional reassurance to the identification hypothesis.^4^ In Section A of Supplementary Materials, we provide further details about the identification and inference concerning the local randomization approach.

### 4.3 Main results

Table 3 presents the main results of the paper. In Panel A, we show the effects of the *EA* on the delay to search for medical care. In panel B, we show the effects of the *EA* on hospitalizations for different bandwidths. In columns (1) to (4), we estimate the sharp regression discontinuity in time specification as shown in Equation (1) for different bandwidths. In column (5), we implement the local randomization approach, shown in Equation (2).

**TABLE 3:** Effects of the *EA* on the demand for medical care

	(1)	(2)	(3)	(4)	(5)
	Sharp regression discontinuity in time				Local randomization
Panel A: Delay to medical care					
Emergency aid	−0.929***	−0.523**	−0.547**	−0.582***	−0.483*
	(0.190)	(0.201)	(0.208)	(0.198)	(0.288)
Control average	6.76	6.76	6.76	6.76	6.76
Bandwidth	5	CCF	12	15	1
Effective *N*	13,110	18,545	33,632	43,141	2740
Panel B: log(Hospitalizations + 1)					
	0.001**	0.001**	0.001	0.001	0.002
	(0.001)	(0.001)	(0.001)	(0.001)	(0.002)
Control average	0.01	0.01	0.01	0.01	0.01
Bandwidth	5	CCF	12	15	1
Effective *N*	320,040	384,048	768,096	960,120	53340

*Note*: This Table shows discontinuity estimates on the demand for healthcare. In columns (1) to (4), we show results for the sharp RDiT for different bandwidths. In column (5), we estimate the local randomization approach. In panel A, the dependent variable is the average time from the first symptoms until testing and, in panel B, it is the log of the number of hospitalizations plus one. We used a uniform kernel. Columns (1) to (4) include weekday and municipality fixed effects. Clustered standard errors at the municipality and day level are in parentheses.

*p < 0.1, **p < 0.05, ***p < 0.01.

The result shown in Table 3 are consistent with our hypothesis. Treatment decreases the lag for medical care by up to 14%, independent of the bandwidth size. Thus, the *EA* allowed individuals to search for the healthcare system and confirm they were infected earlier. Our back‐of‐envelope calculations, displayed in Appendix E, suggest that the *EA* reduced daily infections by approximately 2450 (i.e., almost 20% of the total number of infections at the time) or approximately 73,000 infections during May 2020.

We also observe a moderate sized increase in hospitalizations, of up to 0.015%. This effect decreases as we increase the bandwidth size, which is consistent with our prediction that the “liquidity effect” of the cash transfer is balanced by the “protection effect” after the initial days of treatment. Our back‐of‐the‐envelope calculations, presented in Appendix E, suggest that the *EA* allowed 400 new hospitalizations. These results suggest that even individuals who had severe symptoms (to the point as to require immediate hospitalization) were constrained not to access the healthcare system.

The local randomization approach yields results similar to our main specification for the sharp RDiT. Point estimates shown in column (5) are very close to the ones estimated in column (1), but they are slightly less precise due to the decrease in size of the sample.

Tables S3 and S4, in the Appendix C of the Supplementary Materials, shows that our results are robust to alternative RDD specifications, such as triangular weights and quadratic polynomials, and to other transformations of the hospitalizations data.

**FIGURE 1:**
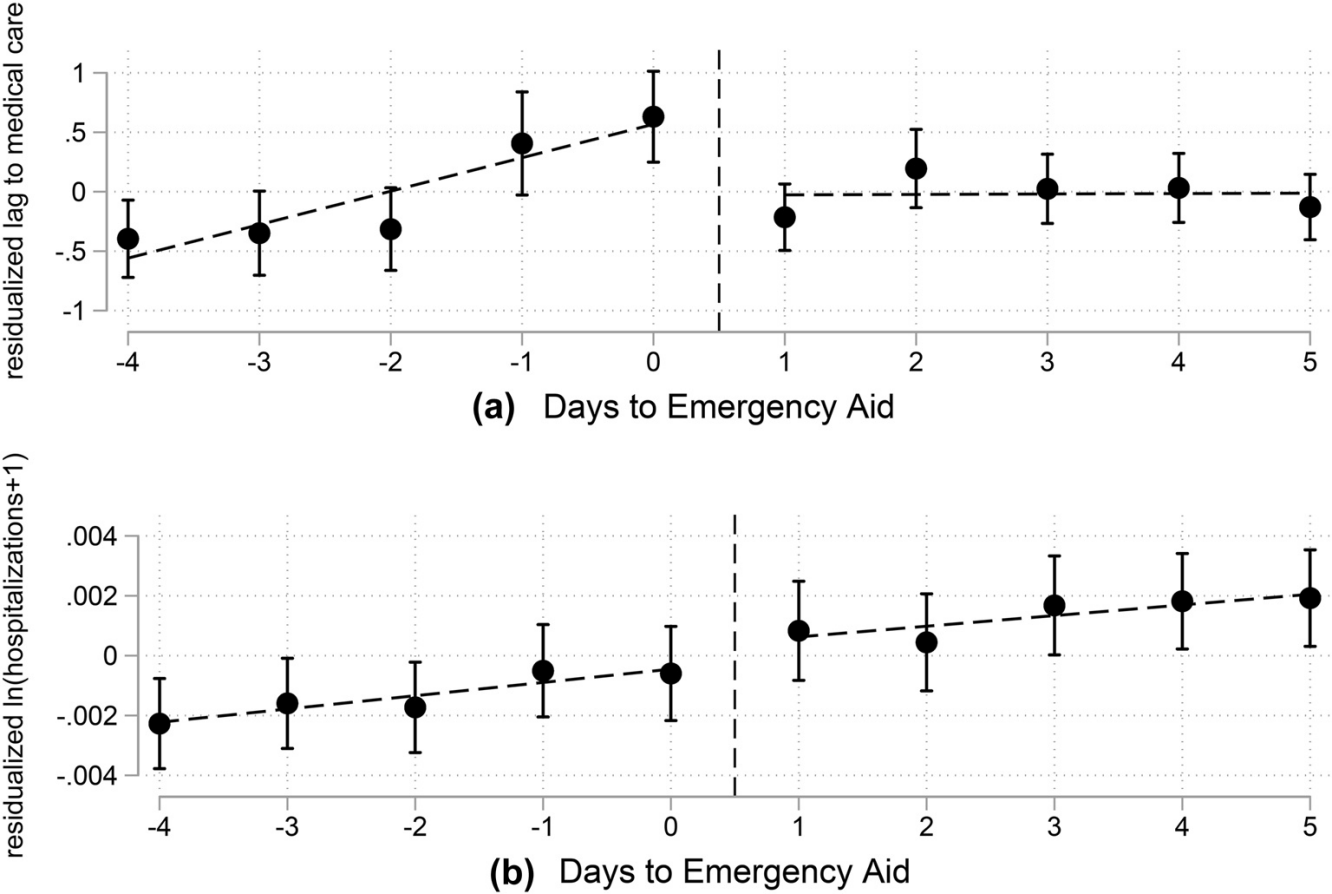
Visual discontinuity evidence. Visual evidence for the discontinuity in the demand for healthcare. The dependent variable is the average time from the first symptoms until testing, in Panel (a), and the log of the number of hospitalizations plus one, in Panel (b) Each bin shows the average and 90% confidence intervals for each bin of the running variable. The dependent variable is residualized by weekday and municipality fixed effects

Figure 1 presents visual evidence of discontinuity in the outcomes of interest. In Figure S1, in Appendix B of the Supplementary Materials, we show RDD figures for the raw data.

### 4.4 Underlying mechanism

Why does the *EA* affects the beneficiaries' healthcare demand? In the two previous sections, we have shown that receiving an income transfer *immediately* increased healthcare consumption. This suggests that the lack of income prevented individuals from seeking healthcare and given that this was a predictable income shock, the beneficiaries' were not able to use credit markets to smooth their healthcare consumption. Therefore, we suggest liquidity constraints is the main underlying mechanism.

However, there are alternative possible interpretations for our findings. First, an income shock might alter the individuals' propensity to incur in risk behavior (Gross & Tabacman, 2014). If this was the case, then the increased demand for healthcare should reflect the changes in particular consumption patterns and not liquidity constraints. We think this interpretation does not fit our application since we focus on very short time horizons after the receiving the transfer, when changes in consumption would not have enough time to affect the need for medical care. We analyze this more formally in Appendix A Supplementary Materials.

Second, our results may have been driven by psychological frictions. Individuals might have “mental accounts” and only spend money from specific income sources on healthcare, such as health insurance, even if they have money available in other “mental accounts” (Thaler, 1985, 1999). In this regard, we build on the recent literature concerning liquidity constraints (Gross et al., 2022; Lyngse, 2020) that examine the effects of income shocks from health insurance. The *EA*, on the other hand, was a general income transfer that was not specifically tied to healthcare and, therefore, it is also very unlikely that the beneficiaries' *a priori* assigned the money to healthcare.

Finally, the cash transfer may have affected the healthcare demand through broader psychological mechanisms. For instance, it might be the case that the lack of money imposes a “psychological tax” on individuals and that positive income shocks increases cognitive function and decision‐making capabilities. However, recent evidence suggests the opposite: the lack of money forces individuals to tunnel their attention and leads to more rational decisions (Fehr et al., 2020; Lichand & Mani, 2020). Thus, if the effect of the *EA* on the demand for healthcare was mediated by this psychological mechanism, we would expect the *EA* to have the opposite effects that we find.

We do not think that any of these alternative explanations are plausible, which only reinforces the interpretation that liquidity constraint is the most important mechanism. To reinforce the confidence in our interpretation, we also show in Table S5, in Appendix C of the Supplementary Materials, direct evidence in favor of the liquidity constraint hypothesis. We show that the increase in the demand for healthcare generated by the *EA* was more intense in municipalities with low access to the financial system, where the beneficiaries were more likely to be liquidity constrained.

## 5 ROBUSTENESS CHECKS

### 5.1 Sorting

Our identifying hypothesis for our favorite specification is that the error‐term is continuous around the cutoff in Equation (1). This identifying assumption is threatened if individual can sort around the cutoff. This is unlikely to have happened, as the government was rigorous in preventing individuals from receiving the money before the due date.

Nonetheless, we can test this using the schedule of payments. Cohorts five and six were paid a week later than others. We can evaluate whether these cohorts already showed any discontinuity in the outcomes before payments were made to them, but after payments were made to the other cohorts. In Table 4, we estimate Equation (1) envisaging that cohorts 5 and 6 received the transfer 6 days before they actually did. We use our favorite bandwidth in this placebo, which does not overlap with the period when these cohorts started receiving the payments.

**TABLE 4:** Effects on the cohorts that have not yet been granted

	Hospitalization	Lag to medical care
Panel A: Cohort 5		
Placebo	−0.002	6.446
	(0.002)	(3.882)
Bandwidth	5	5
Panel B: Cohort 6		
Placebo	−0.001	−0.229
	(0.003)	(0.685)
Bandwidth	5	5

*Note*: Sharp regression discontinuity in time estimates. We adjusted the granting days for cohorts five and six, for 6 days before they actually received the transfer. All estimates use our favorite specification, with 5‐day bandwidth, uniform kernel and a linear polynomial at each side of the cutoff. All estimates include municipality and weekday fixed‐effects. Clustered standard errors at the municipality and day level are in parentheses.

*p < 0.1, **p < 0.05, ***p < 0.01.

We do not find any significant discontinuities for either variable or cohort, reinforcing our reliance on the absence of relevant sorting patterns. In the Appendix D, we discuss and reject other threats to identification, such as spillovers and spurious trends in the outcomes of interest.

### 5.2 Exogenous covariates

Another way to gauge the plausibility of the identifying assumption is to focus on exogenous variables, that should not be affected by the *EA* (Cattaneo et al., 2019). One such variable is the number of COVID‐19 related deaths. Due to the large lag between COVID‐19 infections, hospitalizations, and deaths, it is highly implausible that the *EA* could affect the number of deaths in the very narrow window we look at. If we found any of such effects, we would likely be capturing spurious trends or seasonality patterns.

In Table 5, we re‐estimate our favorite specification, a sharp RDiT with 5 days bandwidth, and our local randomization approach using the number of COVID‐19 related deaths as the outcome of interest.

**TABLE 5:** Effects of the *EA* on the number of Covid‐19 related deaths

	Sharp RDiT	Local randomization
Cash transfer	0.041	−0.067
	(0.085)	(0.060)
*N*	2831	572
Control average	2.80	2.80

*Note*: Discontinuity estimates of the effects of *EA* on the total number of Covid‐19 related deaths. The first column shows an RDiT estimate with 5‐day bandwidth, uniform kernel and a linear polynomial at each side of the cutoff. This estimate also include municipality and weekday fixed‐effects. The second column show local randomization estimates. Clustered standard errors at the municipality and day level are in parentheses.

*p < 0.1, **p < 0.05, ***p < 0.01.

As expected, we find no significant discontinuity in the number of total deaths. If we focus only on a 1‐day window around the cutoff, we also do not find any significant difference in average deaths after the cash transfer is handed out to beneficiaries.

In the Appendix D of Supplementary Materials, we implement an additional robustness test. In Figure S2, we randomly assign cutoff dates to each cohort and estimate placebo treatment effects using our main specification. So, we used this vector of dates and estimated the effects of placebo treatment on our variables of interest. We show that these placebo estimates cannot replicate the true estimates that we discuss above.

## 6 CONCLUSION

In this article, we evaluated the hypothesis that income and liquidity constrained individuals might not be able to seek medical care when they are ill. We explore a cash‐transfer program in Brazil, the Emergency Aid, and its payment calendar that handed the benefit of the program to different cohorts at different times. Using a RDiT, we estimated that a cash transfer increased COVID‐19 hospitalizations by 0.015% and decreased the lag in searching for medical care by about 14%.

The results concerning the lag in searching for medical assistance are robust for several bandwidths. For hospitalizations, the effect diminishes when we increase the bandwidth, which is consistent with our prediction that the “liquidity effect” of the cash transfer is balanced by the “protection effect” after the initial days of treatment.

Previous research has pointed out that individuals delayed healthcare because they were unable to pay small co‐payments, of 2 US$ to 5 US$ (Gross et al., 2022). We focus on public hospitalizations, in which case individuals do not have to incur in any monetary costs. Thus, our results suggest that even the opportunity costs of having to forgo their daily earnings might force individuals to delay seeking medical care.

We also have considered and discussed other possible mechanisms besides the liquidity constraint argument for which the *EA* could affect the health care demand. However, we argue that other potential explanations are not plausible in our context. Even more, we show that effects are stronger in municipalities where individuals are more likely to be liquidity constrained.

## CONFLICT OF INTEREST

Carlos Alberto Belchior and Yara Gomes have nothing to disclose.

## ETHICS STATEMENT

This paper only used publicly available data aggregated at the municipality level. Therefore, ethical approval is not applicable for this article.

## PATIENT CONSENT STATEMENT

This paper only used publicly available data aggregated at the municipality level. Therefore, patient consent is not applicable for this article.

## PERMISSION TO REPRODUCE MATERIAL FROM OTHER SOURCES

This article makes no use of any previously published material.

## CLINICAL TRIAL REGISTRATION

This article does not involve a clinical trial. Therefore, clinical trial registration is not applicable.

## ORIGINAL PUBLICATION STATEMENT

A previous version of this study appeared in **Covid Economics**, issue 80, p. 78–93, as a pre‐print. The authors still retain copyright.

## Supporting information

Supporting Information S1

## Data Availability

The data that support the findings of this study are available from the corresponding author upon reasonable request.

## ENDNOTES

^1^
The value of the transfer was reduced by half during 2021.

^2^
This is the reason we consider the total number of hospitalizations in each municipality and we do not need to adjust for population. Formally, using per capita hospitalizations as a dependent variable with a logarithmic specification is equivalent to adding the log of the population in the right side of Equation (1). Since the population does not vary within municipalities for the small time‐horizon used in the regressions, this term is not separately identified. Thus, using total or per capita hospitalizations yields the same estimator.

^3^
We use the two‐way clustering procedure suggested by Cameron, Gelbach, and Miller (​**2011**).

^4^
The need to include covariates in the RDiT design is very common in the literature. We surveyed eight recent papers that rely on this design and were recently published in top economics journals and all but one of them included some type of covariate to control for seasonality. This is shown in Table S1 in Appendix A of Supplementary Materials.
