# Supplementary material for: Liquidity constraints, cash transfers and the demand for health care in the Covid‐19 pandemic
Source: Health Econ. 2022 Aug 22;31(11):2369–80. doi: 10.1002/hec.4585 (PMC9538205; doi:10.1002/hec.4585)
Supplement: Supplementary file 1 — Supporting Information S1 [file HEC-31-2369-s001.pdf]

Supplementary Materials
Liquidity constraints, cash transfers and the demand for health care in the Covid-19 pandemic

July 17, 2022

In this Supplementary Material, we provide additional results of the paper "Liquidity constraints, cash transfers and the demand for health care in the Covid-19 pandemic". Appendix A discusses additional technical details that include time-varying treatment effects, bandwidth choice, the inclusion of covariates, and estimation details for the local randomization approach. Appendix B shows additional descriptive statistics. Appendix C shows additional results. First, we show that the main results are robust to variations in the specification choice. Second, we provide ancillary descriptive evidence in favor of the liquidity constraint hypothesis. Appendix D discusses other threats to identification and additional robustness checks. Appendix E shows back-of-the-envelope calculations for the magnitudes of the effects of the Emergency Aid on hospitalizations and decrease in transmissions.

A. Additional technical details

Specification discussion

One specificity of the RDiT framework is that the implementation requires the specification of how the treatment effect varies according to time. Hausman and Rapson (2018) show that incorrectly specifying these treatment effects might bias the results. In our context, this is intrinsically linked to bandwidth choice.

Most recent studies that implemented this methodology focused only on very short-run treatment effects or assumed time-invariant treatment effects. In the next subsection, we show a survey using the RDiT methodology. On the other hand, our application allows us to make stricter predictions about the time-varying treatment effects of the cash transfer on hospitalizations.

Consider the following timeline for infected and non-infected individuals, respectively:

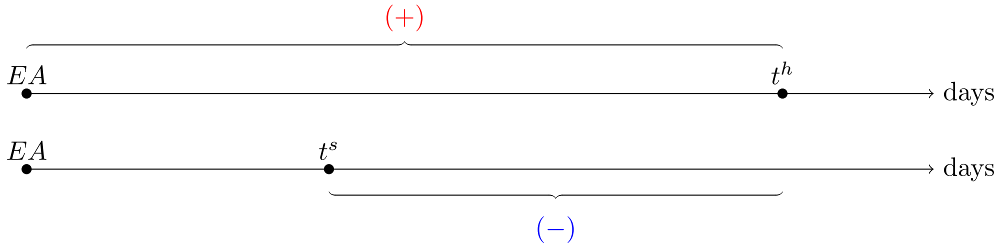

The cash transfer has two immediate effects on beneficiaries. It alleviates liquidity constraints, which we call the "liquidity effect", and it allows individuals to social distance and protect themselves against the pandemic, which we call the "protection effect". Conditional to being infected, the "liquidity effect" increases hospitalizations immediately. On the other hand, the "protection effect" decreases the probability that individuals get infected and need hospitalizations.

Thus, when both the "liquidity" and the "protection" effects are at play, we cannot identify the hospitalizations response to the EA. However, we can take advantage of the different timings of these effects. If individuals start protecting themselves as soon as they receive the cash transfer, this will only decrease hospitalizations with a lag, determined by the Sars-CoV-2 incubation period or the lag between being infected and experiencing the first symptoms (t^s), which is close to five days (BACKER et al., 2020).

We expect that the effect on hospitalizations will be positive in the first five days after treatment since only the "liquidity effect" is at play. Once we consider larger time horizons, the effect on hospitalizations becomes uncertain. That is consistent with our empirical estimates presented in Table 3. We find a positive and statistically significant treatment effect for windows of five days or less. Once we increase the bandwidth and give weight to observations beyond that time horizon, we estimate closer to zero and statistically non-significant results.

Regarding the other outcome of interest, the lag to search for medical care, we expect treatment effects not to be heterogeneous relative to time. Consistent with that, our estimates show that treatment effects for this other variable tend to be less sensitive to the bandwidth choice.

Another important part of our empirical design is the inclusion of weekday covariates to control seasonality in the dependent variable. We argued in the main text that the inclusion of these covariates increases the plausibility of the identification hypothesis. In the next subsection, we discuss the prevalence of this strategy in the literature.

Comparisons to the literature

In the previous subsection, we compared our empirical specification with the literature in two respects. First, we argued that our context allows a more strict prediction about time-heterogeneity than other papers with a similar methodology. Second, we argued that the inclusion of covariates is prevalent. We surveyed eight papers that used this methodology and were published in top economics journals. In Table S.1, we tabulate how they dealt with time-heterogeneity and the use of covariates.

Table S.1: Survey of the literature

	Setting	Time-heterogeneity	Time fixed-effecs
Anderson (2014)	Traffic	Discuss short x long run	day of the week
Auffhammer and Kellogg (2011)	Air quality	Only short-run	No
Bento et al. (2014)	Traffic	Only short-run	month x day, and hours
Busse et al. (2006)	Vehicle prices	Only short-run	week
Davis (2008)	Air quality	Discuss short x long run	month, day of the week, hour
De Paola et al. (2013)	Car accidents	Only short-run	month, day of the week, holidays
Gallego et al. (2013)	Air quality	Only short-run	Hour, day of the week, month
Ouss (2020)	Juvenile incarceration	Only short-run	Month x day trend

Two interesting facts arise from the descriptive table above. First, all but two papers only estimate short-run treatment effects. Two others broadly discuss the potential differences between short and long-run treatment effects. Our paper is the first one in the literature to provide strict predictions about these differences to the best of our knowledge. Second, seven out of the eight papers rely on some time fixed-effects to increase the plausibility of the identification hypothesis.

Local randomization approach

In this subsection, we discuss identification and inference for the local randomization approach. In the main text, we implement this approach in column (5) of Table 3. In this approach, we assume that the assignment of treatment is random within a narrow window around the cutoff. Other than the usual assumptions, this implies that potential outcomes are unrelated to the running variable.

To make this hypothesis more plausible, we select the smallest possible window - only one day before and after the transfer. By focusing on such narrow bandwidth, we expect the seasonality of the dependent variable to be limited. However, one persistent potential problem is that the outcomes reported on the weekend are very different from other days. In order to account for this, we exclude the first cohort (that received the transfer in a Monday) from the sample. Table 5 shows that deaths (that we consider to be predetermined) do not vary in this narrow window.

This estimation approach usually requires additional specifications. The kernel specification is irrelevant because of the few data points at each cutoff side. Our implementation also considers a fixed-margin treatment assignment mechanism instead of a Bernoulli process.

Also, inference for the local randomization approach is not straightforward. Young (2019) shows that asymptotic inference methods of calculating standard errors fail in experiments, especially in the ones with a small number of observations. Focusing on only two days of the sample, we severely restrict the effective sample size (especially for the lag to medical care, which is not available

to all municipalities). Thus, we calculate exact p-values using a randomization inference.

B. Additional descriptive statistics

In the main text, we show RDD figures residualizing outcome variable by weekday and municipality fixed-effects. We argued that these fixed effects are necessary to control for seasonality patterns. In Figure S.1, we show RDD figures using raw data.

Figure S1: Raw outcome averages for each value of the running variable

Panel A: Lag to medical care

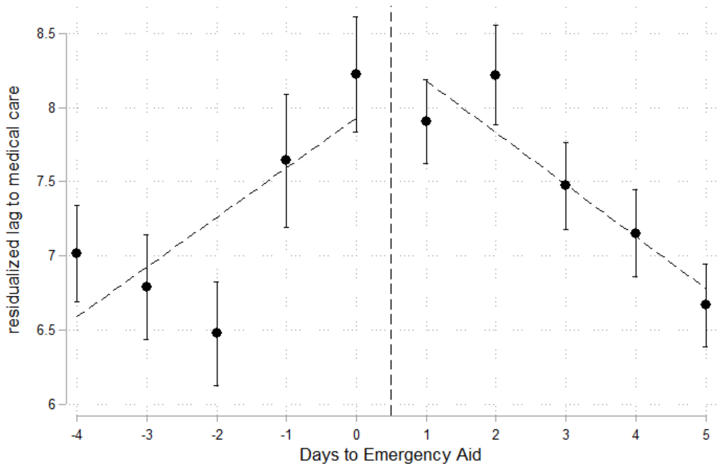

Panel B: Hospitalizations

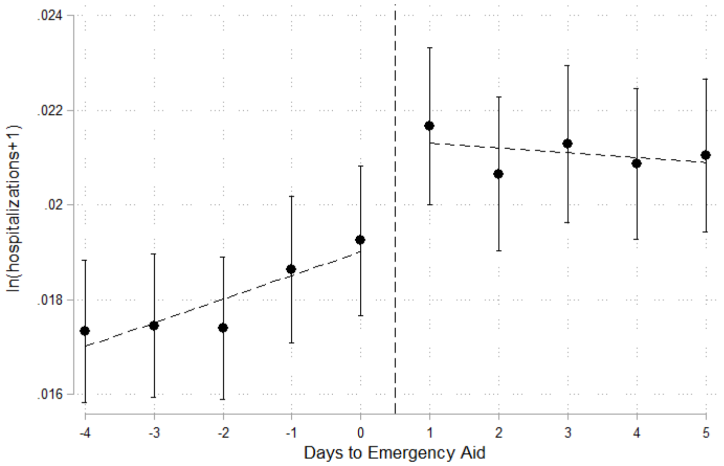

Note: Visual evidence for the discontinuity in the demand for healthcare. The dependent variable is the average time from the first symptoms until testing, in Panel A, and the log of the number of hospitalizations plus one, in Panel B. Each bin shows the average and 90% confidence intervals for each dependent and value of the running variable.

We can see the discontinuity for hospitalizations is almost identical to the residualized version. The raw data for the lag to search for medical care, on the other hand, does not suggest a clear discontinuity. Since there is a large seasonality in the lag to search for medical care, as we argued above, the interpretation of the raw data is not straightforward. Also, the fact that we find consistent negative effects of the *EA* on the lag to medical care using both the sharp RDiT and the local randomization approach suggests that these raw figures are not the best way to evaluate the causal effects of the program.

Next, our interpretation of the data for hospitalizations is that is provides a measure of liquidity sensitivity for individuals with severe symptoms. To provide additional context, we show in Table S.2 some individual-level descriptive statistics for the hospitalizations sample.

Table S.2: Descriptive statistics for hospitalized individuals

	average	standard-deviation	min	max
**Panel A**: Individual characteristics				
Male	0.56	0.50	0.00	1
White	0.41	0.49	0.00	1
Age	59.23	18.55	0.00	100
Elderly	0.42	0.49	0.00	1
**Panel B**: Medical conditions				
Any Risk factor	0.65	0.48	0.00	1
Heart condition	0.18	0.39	0.00	1
Liver condition	0.38	0.49	0.00	1
Renal condition	0.36	0.48	0.00	1
Asthma	0.37	0.48	0.00	1
Diabetes	0.23	0.42	0.00	1
Imunodepressed	0.37	0.48	0.00	1
Obesity	0.34	0.48	0	1
**Panel C**: Medical consequences				
Intensive care	0.35	0.48	0.00	1
Fever	0.58	0.49	0.00	1
Cough	0.23	0.42	0.00	1
Sore throat	0.17	0.38	0.00	1
Diarrhea	0.57	0.49	0.00	1
Vomit	0.61	0.49	0.00	1
Abdominal pain	0.65	0.48	0.00	1
Smell loss	0.18	0.39	0.00	1
Taste loss	0.17	0.37	0.00	1

Note: This Table averages, standard-deviation, minimum and maximum values for several variables. We include in the sample individuals that were hospitalized due to Covid-19 in the public system during the period of analysis.

These descriptive statistics reinforce our interpretation of the hospitalizations data. Hospitalized individuals have advanced age (more than 42% were elderly individuals), 65% of individuals presented some risk factor related to *Covid*-19, and 35% required intensive care.

C. Additional results

Alternative specifications

Our favorite specification in the main text uses a five-day bandwidth, a linear polynomial on each side of the cutoff, and a uniform kernel. Table 3 shows how our results varied according to bandwidth choice. In Table S.3, we show results for other specifications. In column (1), we change the kernel to a triangular one. In column (2), we use a quadratic polynomial at each side of the cutoff, and, in column (3), we implement a difference-in-difference strategy instead of regression discontinuity in time. We can see that results are similar to those in the main text.

Table S.3: Effects of the *EA* on healthcare consumption with alternative specifications

	Regression discontinuity in time		Difference-in difference
Panel A: Delay to medical care			
Treatment	-0.958***	-1.334***	-0.565***
	(0.1964)	(0.3306)	(0.1578)
Control average	6.76	6.76	6.76
Effective N	13,110	13,110	26,022
Panel B: log(Hospitalizations+1)			
Treatment	0.001**	0.001*	0.002***
	(0.0004)	(0.0005)	(0.0004)
Control average	0.01	0.01	0.01
Effective N	320,040	320,040	1,152,144
Bandwidth	5	5	-
Kernel	triangular	uniform	-
Polynomial	linear	quadratic	-

Note: Estimates of a regression discontinuity in time (columns 1 and 2) and difference-in-difference strategy. In panel A, the dependent variable is the average time from the first symptoms until testing and, in panel B, it is the log of the number of hospitalizations plus one. Clustered standard-errors at the municipality and day level are presented in parentheses. * p<0.1, ** p<0.05, *** p<0.01.

In the main text, when analyzing the effect of the emergency aid on hospitalizations, we transformed the dependent variable using the log of hospitalizations plus one. We argued that this allowed us to interpret coefficients as approximate semi-elasticities and did not require us to drop observations without hospitalizations. However, Bellamare and Wichman (2020) show that this type of *ad hoc* transformation may substantially differ from the desired elasticity.

In Table S.4, we show that our results are not sensitive to the chosen transformation. We report the effects of the *EA* on the total number of hospitalizations (column 1), log of hospitalizations plus 0.1 (column 2), log of hospitalizations plus 0.01 (column 3), and the inverse hyperbolic sine transformation (column 4). The results are similar to the ones shown in the main text. Across specifications, we find positive and small effects of the *EA* on hospitalizations. In columns (2)-(4), we test the hypothesis that the estimated coefficients, which

can also be interpreted as approximate semi-elasticities, are equal to our favorite specification reported in the main text. We cannot reject any of the three null hypotheses. Finally, if anything, our preferred choice of transformation is somewhat conservative since all semi-elasticities shown in Table S.4 are higher than the ones obtained in the main text.

Table S.4: Effects of the *EA* on hospitalizations with alternative transformations

	(1)	(2)<br>(h+0.1)	(3)<br>(h+0.01)	(4)<br>(h+$\sqrt{h^2+1}$)
Treatment	0.004**	0.004	0.006	0.001**
	(0.0016)	(0.0027)	(0.0041)	(0.0006)
Equal to (h+1)<br>p-value	-	0.29	0.28	0.64
Control average	0.04	-4.55	-6.82	0.01
Bandwidth	5	5	5	5
Effective N	320,040	320,040	320,040	320,040
Weekday fixed-effects	yes	yes	yes	yes
Municipality fixed-effects	yes	yes	yes	yes

Note: This Table shows sharp RDiT estimates of the effect of the *EA* on different measure of hospitalizations. We used a uniform kernel, five-day bandwidth and linear polynomial at each side of the cutoff. All columns include weekday and municipality fixed effects. Clustered standard errors at the municipality and day level are in parentheses. * p<0.1, ** p<0.05, *** p<0.01.

Liquidity constraint hypothesis

In the main text, we found that the *EA* immediately increased beneficiaries' demand for healthcare. We argued in the main text that beneficiaries' were likely to be income- and liquidity-constrained not to seek healthcare. That is, individuals' low income prevented them from demand healthcare, and liquidity restrictions prevented them from borrowing money.

However, we also pointed out that potential alternative mechanisms might explain our reduced-form results. This section of the Appendix presents direct descriptive evidence in favor of the liquidity constraint hypothesis.

The theoretical literature on liquidity constraints has traditionally emphasized that asymmetric information and transactional costs could generate borrowing constraints (Wilcox, 1989). We explore heterogeneity in the latter to provide descriptive evidence in favor of the liquidity constraint hypothesis.

We collect data for the geographical distribution of banking agencies from the Brazilian Central Bank. Then, we calculate the number of banking agencies in each municipality relative to the population. Individuals in Brazil usually have to attend banking agencies to obtain formal loans.

For that reason, individuals who live in municipalities with a small number

of banking agencies (or even no agencies) are likely to face higher transactional costs to borrow and are more likely to be liquidity constrained. Thus, we expect the *EA* to have larger effects on individuals that are far from banking agencies.

We split our sample into municipalities below and above the median of banking agencies. Then, we estimate our favorite specification for the sharp RDiT for each subsample. Results are shown in Table S.5:

Table S.5: Effects of the *EA* on demand for medical care by municipality access to the financial system

	Low access to financial system	High access to financial system
**Panel A**: Delay to medical care		
Cash transfer	-1.5874***	-0.5377*
	(0.2258)	(0.2857)
Bandwidth	5	5
Observations	5,049	8,061
**Panel B**: log(Hospitalizations+1)		
Cash transfer	0.0013***	0.0008
	(0.0003)	(0.0009)
Bandwidth	5	5
Observations	160,020	160,020

Note: This Table shows sharp RDiT estimates of the effect of the *EA* on the demand for medical care. In panel A, the dependent variable is the average time from the first symptoms until testing and, in Panel B, it is the log of the number of hospitalizations plus one. In the first column, we restrict the sample to municipalities with below median access to the financial system. In the second column, we restrict the sample to municipalities with above median access. We used a uniform kernel, five-day bandwidth, and a linear polynomial at each side of the cutoff. All regressions include weekday and municipality fixed effects. Clustered standard errors at the municipality and day level are in parentheses. * p<0.1, ** p<0.05, *** p<0.01.

We find that the demand for medical care, measured both as the lag to search for healthcare and the number of hospitalizations, is higher in municipalities with low access to the banking sector than in those with better access. Of course, the distribution of banking agencies is not random and these results might be explained by unobserved factors that correlate with the demand for medical care and the geographical distribution of banking agencies. Nonetheless, in our view, a combination of the unlikelihood of the alternative explanations and this ancillary evidence paints a clear picture in favor of the liquidity constraint hypothesis.

D. Threats to identification and robustness checks

The fundamental identification hypothesis in our empirical analysis is that the error term distribution is continuous around the cutoff. In this case, β in equation (1) of the main text recovers the causal effect of EA on the variable of interest. Intuitively, we need to assume that non-observable confounders do not change discontinuously when the EA is given.

There are possible threats to this identifying assumption. In the main text, we discussed the threat of sorting around the cutoff. A second concern is that there might be within-family spillovers. The day the cash transfers are handed out to families is determined by the birth cohort of the person of reference in the family. Since the transfer increases liquidity for all family members, it might affect individuals of different birth cohorts. A third concern is that our empirical framework confounds spurious trends related to the dynamics of the pandemic as discontinuities.

In section 4.4 of the main text, we showed that there were no discontinuities in the days before the cohorts actually received the transfer for the cohorts would only receive the payment six days later. This indicates that: 1) cohorts could not sort around the cutoff and receive the payment earlier, and 2) that one cohort receiving payment does not have an observable spillover to the other.

We test the plausibility of the third threat to the identifying assumption by randomly assigning cutoff dates and estimating placebo treatment effects using our main specification. That is, we drafted a vector of six fictitious treatment dates $\overline{\boldsymbol{EA}}_c$ and assigned them to each of the six cohorts in the sample. So, we used this vector of dates and estimated the effects of placebo treatment on our variables of interest.

We repeated this procedure 500 times and recorded the associated z statistics for each one. We expect not to be able to reproduce z statistics as extreme as the true ones with these placebos. In Figure S.1, we present the estimated distribution of placebos:

Figure S.2: Distribution of z statistics for placebos

Panel A: Placebos for hospitalizations

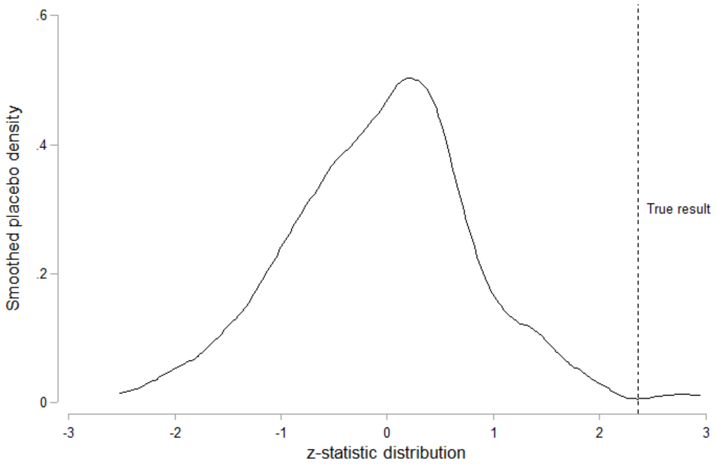

Panel B: Placebo for time for reaching medical care

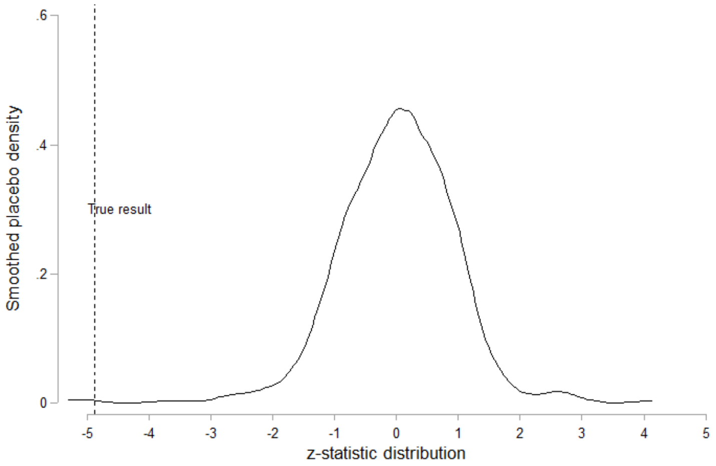

Note: Smoothed distribution of the 500 z statistics obtained with for $\overline{\boldsymbol{EA}}_c$ drafts. In Panel A, the dependent variable is the number of hospitalizations and, in Panel B, the time to seek medical care.

The estimated z statistics are centered around zero. For the lag of medical care, 2 of the 500 drafts that were carried out had z statistics as extreme as the true result (p-value of 0.004). For hospitalizations, 14 of the 500 drafts that were carried out had z statistics as extreme as the true results (p-value 0.028).

E. Back-of-the-envelope calculations

In this section, we try to gauge the magnitudes of the effects of the EA on hospitalizations and subsequent infections. First, we calculate the effect of the EA on hospitalizations, which is straightforward. Let β^h be the estimated percentage increase in hospitalizations shown in Table 3. Also, let $\overline{H}$ be the average hospitalizations at the municipality/day-level in 2020. Then, the magnitude of the effect on hospitalizations can be calculated by:

$$\Delta H = \beta^h * \overline{H} * M * h$$

where M is the total number of Brazilian municipalities, and h is the used bandwidth of five days. Making this simple calculation, we concluded that the EA allowed 400 new hospitalizations in the five days following the transfer.

The increased demand for health care may not only increase the welfare of beneficiaries but also have positive externalities. For instance, if individuals are aware sooner that they contracted the Sars-CoV-2 virus, they might be less prone to infecting others. Now, we provide back-of-the-envelope calculations of the size of this reduction in infections.

The timeline of the contraction and evolution of the disease can be represented below:

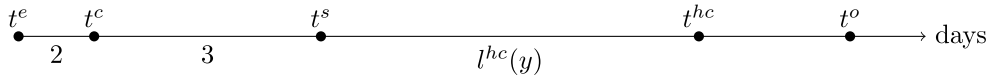

First, individuals are exposed to the virus (t^e). They can transmit the disease two days later (t^c). This happens before the appearance of symptoms (t^s) that only happens five to seven days after exposure. After the appearance of the first symptoms, individuals may search for the healthcare system, which happens at (t^{hc}). Throughout the paper, we focused on the role of cash transfer (y) in reducing the lag to search for health care (l^{hc}). Finally, after some time, the disease has an outcome (t^o).

Let c_{it} be a random variable that indicates number of persons that an individual i that contracted the disease in the period t transmits the disease. The expectancy of total contagions is $E(c_{it}) = R_t$, for an individual exposed in t.

In order to do the back-of-the-envelope calculations, we make the simplifying assumption that $c_{it} \sim U[t_i^c, t_i^{hc}]$. That is, we assume that individuals have an equal probability of infecting others since t^c until they are diagnosed (t^{hc}). Then, expected daily infections is given by $E(c_{it}) = \frac{R_t}{\bar{t}^{hc}(y) - \bar{t}^c}$. Thus, we can calculate the decrease in infections (ΔI) by:

$$\Delta I = \sum_i \frac{\partial t^{hc}}{\partial y} \frac{R_t}{\bar{t}^{hc} - \bar{t}^c}$$

To calculate ΔI, we use the cash transfer effect on the delay to seek medical care estimated in Table 3 (column 1) and averages for the lapse between

symptoms and testing in Table 2. In May 2020, the Brazilian Covid-19 reproduction rate was calculated to be 2.91 (Doornik et al., 2020) and an average of approximately 12,500 new daily cases.

Plugging those values into equation (2), we calculate that the cash transfer decreased by ≈ 2450 the number of daily new Covid-19 cases. This amounts to a decrease of $\approx 73,000$ Covid-19 in May 2020. This calculation considers only the first-order decrease in cases generated by the cash transfer. We do not consider that individuals who were not exposed due to the treatment would expose other individuals to the disease (second-order effects).

References

Auffhammer, Maximilian and Ryan Kellogg (2011). Clearing the Air? The Effects of Gasoline Content Regulation on Air Quality. *American Economic Review*, vol.101, n.6, 2687–2722.

Anderson, Michael L (2014). Subways, Strikes, and Slowdowns: The Impacts of Public Transit on Traffic Congestion. *American Economic Review*, vol.104, n.9, p.2763–2796.

Backer, Jantien A; Don Klinkenberg, and Jacco Wallinga (2020). Incubation period of 2019 novel coronavirus (2019-nCoV) infections among travellers from Wuhan, China, 20–28 January 2020. *Europe's journal on infectious disease surveillance, epidemiology, prevention and control*, vol.25, n.3.

Bellamare, Marc and Casey J. Wichman (2020). Elasticities and the Inverse Hyperbolic Sine Transformation. *Oxford Bulletin of Economics and Statistics*, vol.82, n.1.

Bento, Antonio, Daniel Kaffine, Kevin Roth, and Matthew Zaragoza-Watkins (2014). The Effects of Regulation in the Presence of Multiple Unpriced Externalities: Evidence from the Transportation Sector. *American Economic Journal: Economic Policy*, vol.6, n.3, p.1–29.

Busse, Meghan, Jorge Silva-Risso, and Florian Zettelmeyer (2006). $1,000 Cash Back: The Pass-Through of Auto Manufacturer Promotions," American Economic Review, vol.96, n.4, p.1253–1270.

Davis, Lucas W (2008). The Effect of Driving Restrictions on Air Quality in Mexico City. *Journal of Political Economy*, vol.116, n.1, p.38–81.

De Paola, Maria, Vincenzo Scoppa, and Mariatiziana Falcone (2013). The Deterrent Effects of the Penalty Points System for Driving Offences: A Regression Discontinuity Approach. *Empirical Economics*, vol.45, p.965–985.

Doornik, Jurgen; Jennifer Castle and David Hendry (2020). Short-term forecasting of the coronavirus pandemic. *International Journal of Forecasting*, 12 September.

Gallego, Francisco, Juan-Pablo Montero, and Christian Salas (2014). The Effect of Transport Policies on Car Use: Evidence from Latin American Cities. *Journal of Public Economics*, vol.107, p.47–62.

Hausman, Catherine e David S. Rapson (2018). Regression Discontinuity in Time: Considerations for Empirical Applications. *Annual Review of Resource*

Economics, vol.10, p.533-552.

Ouss, Aurélie (2020). Misaligned incentives and the scale of incarceration in the United States. *Journal of Public Economics*, vol.191.

Wilcox, James (1989). Liquidity Constraints on Consumption: The Real Effects of "Real" Lending Policies. *Economic Review*, n.4, p.39-52.

Young, Alvin (2019). Channeling Fisher: Randomization Tests and the Statistical Insignificance of Seemingly Significant Experimental Results. *Quarterly Journal of Economics*, vol.134, n.2.
